# *Salmonella* ubiquitination: ARIH1 enters the fray

**DOI:** 10.15252/embr.201744672

**Published:** 2017-08-18

**Authors:** Damián Lobato‐Márquez, Serge Mostowy

**Affiliations:** ^1^ Section of Microbiology MRC Centre for Molecular Bacteriology and Infection Imperial College London London UK

**Keywords:** Microbiology, Virology & Host Pathogen Interaction, Post-translational Modifications, Proteolysis & Proteomics

## Abstract

Ubiquitination is a post‐translational modification in which ubiquitin, a 76‐amino acid polypeptide, is covalently bound to one or more lysines of a target protein. Ubiquitination is mediated by the coordinated activity of ubiquitin activating (E1), conjugating (E2), and ligating (E3) enzymes. Ubiquitin is widely investigated for its ability to regulate key biological processes in the cell, including protein degradation and host–bacteria interactions. The determinants underlying bacterial ubiquitination, and their precise roles in host defense, have not been fully resolved. In this issue of *EMBO Reports*, Polajnar *et al* 1 discover that Ring‐between‐Ring (RBR) E3 ligase ARIH1 (also known as HHARI) is involved in formation of the ubiquitin coat surrounding cytosolic *Salmonella*. Evidence suggests that ARIH1, in cooperation with E3 ligases LRSAM1 and HOIP, modulates the recognition of intracellular bacteria for cell‐autonomous immunity.

*Salmonella enterica* is a Gram‐negative bacterial pathogen and major causative agent of gastroenteritis worldwide. Among the *S. enterica* serovars, Typhimurium has emerged as a convenient model organism to study the cellular response to bacterial infection because its infection process has been well defined. For host cell invasion and intracellular survival, *S*. Typhimurium employs two type III secretion systems to secrete effector proteins and manipulate cellular processes. Intracellular bacteria reside in the *Salmonella* containing vacuole (SCV), where they prevent phagolysosome fusion and replicate. However, during infection of epithelial cells, a fraction of *Salmonella* escape from the SCV to the cytosol, where bacteria are decorated by a ubiquitin coat and targeted to autophagy, an intracellular degradation process. Landmark studies have used cytosolic *S*. Typhimurium to discover the recruitment of ubiquitin and ubiquitin‐binding autophagy receptors p62, NDP52, and OPTN to bacteria for their selective clearance via autophagy 2, 3, 4. Collectively, a picture emerges that ubiquitination of cytosolic bacteria and its recognition by autophagy is a carefully coordinated process crucial for host defense.

How are cytosolic bacteria recognized by ubiquitin? Systematic ubiquitination site profiling revealed that *Salmonella* outer membrane proteins (OMPs) can be ubiquitinated 5, but the E3 ligases that ubiquitinate bacteria remain largely elusive. Studies have shown that E3 ligases LRSAM1, RNF166, and HOIP can mediate the recruitment of ubiquitin and ubiquitin‐binding autophagy receptors to cytosolic *Salmonella* 6, 7, 8. However, LRSAM1 is only partially responsible for the *Salmonella* ubiquitin coat, RNF166 mediates ubiquitination of p62 and not bacteria per se, and the recruitment of HOIP requires an upstream E3 ligase. Therefore, these E3 ligases appear not to be sufficient to eliminate cytosolic bacteria. Considering that PARKIN, a member of the RBR E3 ligase family, has been shown to mediate ubiquitination of bacterial pathogens including *Mycobacterium tuberculosis* 9, Polajnar *et al* 1 screened all 14 members of the RBR E3 ligase family for their ability to ubiquitinate *Salmonella* and restrict their proliferation. Employing an RNA interference (RNAi) library and a *Salmonella* ∆*sifA* mutant engineered to fluoresce upon exposure to the cytosol (the bacterial effector SifA is required for SCV integrity), the authors identified ARIH1. The specific pathways regulated by ARIH1 are poorly understood, and a role for ARIH1 in bacterial ubiquitination was unknown. Experiments revealed ARIH1 co‐localization with ubiquitinated *Salmonella* and an increase in the number of cytosolic bacteria in ARIH1‐depleted cells. In agreement with a role for ARIH1 in the restriction of bacterial proliferation, LRSAM1 is recruited to ARIH1‐positive bacteria. To determine how ARIH1 modifies cytosolic bacteria, the authors performed immunofluorescence microscopy and *in vitro* ubiquitination assays using purified components. These experiments showed that ARIH1 decorates cytosolic bacteria with K48‐linked ubiquitin chains in a process dependent on *Salmonella* OMPs. *In vitro*, ARIH1 activity depends on its interaction with neddylated cullin‐RING ligases. Interestingly, ubiquitination of bacteria by ARIH1 is independent of neddylated cullin‐RING ligases; thus, further work is needed to understand the molecular basis of ARIH1 activation during *Salmonella* infection.

To test whether ARIH1 ubiquitinates bacteria for degradation by autophagy, the authors depleted ARIH1 from cells with or without ATG7, the activating enzyme for LC3B conjugation. The depletion of ARIH1 from ATG7 knockout cells led to a significant increase in the number of cytosolic bacteria, suggesting that ARIH1 ubiquitination can protect host cells by a mechanism independent of autophagy. Consistent with this, depletion of ARIH1 does not affect the co‐localization of autophagy markers p62, NDP52, OPTN, or LC3B to cytosolic bacteria. These results demonstrate that ubiquitination by ARIH1 is antibacterial, yet different than ubiquitination by LRSAM1 which is required for autophagy marker recruitment 7. However, in cells depleted for both ARIH1 and LRSAM1, the authors did not detect a significant increase in total bacterial burden as compared to cells depleted for ARIH1 or LRSAM1 alone. These data suggest that ARIH1 and LRSAM1 can function in the same antibacterial pathway.

Surprisingly, the depletion of ARIH1 and/or LRSAM1 was shown to trigger linear ubiquitination of cytosolic *Salmonella* and the activation of NF‐κB signaling. Other studies have recently shown that linear ubiquitination can restrict bacterial proliferation by activating NF‐κB 8, 10. These new data indicate that linear ubiquitination can compensate for the loss of ARIH1 and LRSAM1. To further investigate the coordination of different E3 ligases during *Salmonella* infection, the authors performed depletion experiments and used super‐resolution microscopy to investigate their recruitment. In agreement with an important role for ARIH1 in the ubiquitination of cytosolic *Salmonella*, the depletion of LRSAM1 or HOIP led to a significant increase in patchlike recruitment of ARIH1 to bacteria that escaped the SCV. Although an interaction between these E3 ligases was not shown, the authors propose that ARIH1, LRSAM1, and HOIP form a network that together serves to restrict the replication of cytosolic bacteria **(**Fig 1). It remains to be fully determined how ARIH1, LRSAM1, and HOIP cooperate with each other to modulate the *Salmonella* ubiquitin coat, and how this cooperation can precisely regulate the host response to infection.

**Figure 1:**
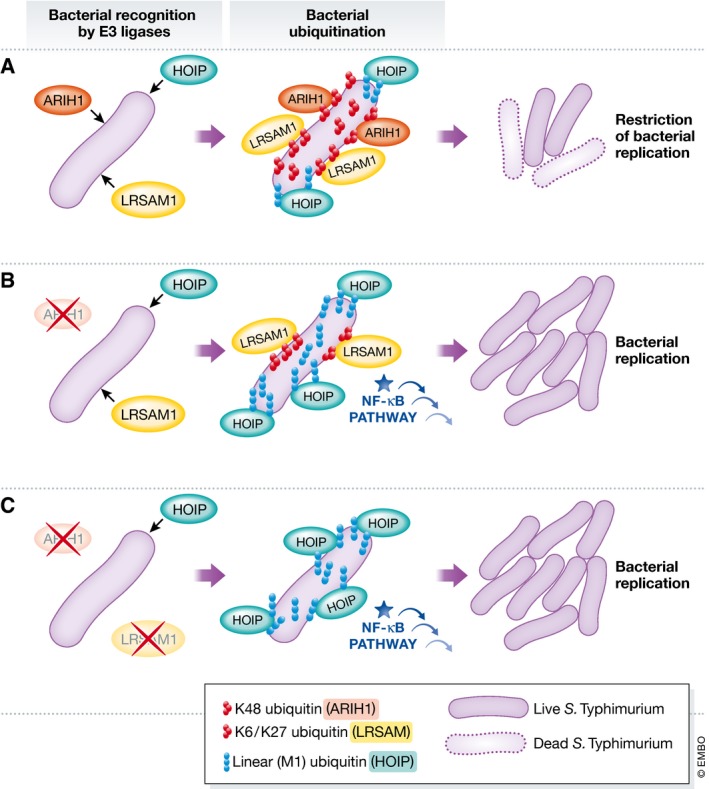
A new role for ARHI1 in host defense against bacterial infection (A) The E3 ubiquitin ligases ARIH1, LRSAM1, and HOIP cooperate to mediate ubiquitination (K48‐, K6‐/K27‐, and M1‐linked, respectively) of cytosolic *Salmonella* and restrict bacterial replication. (B) In the absence of ARIH1, LRSAM and HOIP mediate ubiquitination (K6‐/K27‐ and M1‐linked, respectively) of cytosolic *Salmonella*. HOIP‐mediated linear ubiquitination is increased, and the NF‐κB signaling pathway is activated. In this case, bacterial replication is not restricted. (C) In the absence of both ARIH1 and LRSAM1, HOIP‐mediated linear ubiquitination is increased and the NF‐κB signaling pathway is activated. In this case, bacterial replication is not restricted.

In summary, the discovery of ARIH1 expands the repertoire of E3 ligases mediating the ubiquitination of cytosolic *Salmonella* for host defense. These results also highlight that ubiquitin can have regulatory roles during infection beyond targeting bacteria to autophagy. While we have a good molecular understanding of *Salmonella* ubiquitination, we still lack insights into the events that take place when cytosolic bacteria are recognized and targeted by ubiquitin. Further studies are required to understand how E3 ligases cooperate with each other, which signaling mechanisms are important for host defense, and how these processes can be manipulated for therapeutic purposes.
